# Metal mobilization from soils by phytosiderophores – experiment and equilibrium modeling

**DOI:** 10.1007/s11104-014-2128-3

**Published:** 2014-06-04

**Authors:** W. D. C. Schenkeveld, E. Oburger, B. Gruber, Y. Schindlegger, S. Hann, M. Puschenreiter, S. M. Kraemer

**Affiliations:** 1 Dept. of Environmental Geosciences Center for Earth Sciences, University of Vienna, Althanstraße 14 (UZA II), 1090 Vienna, Austria; 2 Department of Forest and Soil Sciences, University of Natural Resources and Life Sciences, Konrad Lorenz Strasse 24, 3430 Tulln, Austria; 3 Department of Chemistry, University of Natural Resources and Life Sciences, Muthgasse 18, 1190 Vienna, Austria

**Keywords:** Phytosiderophores, Fe acquisition, DMA, Fe shuttle, Metal mobilization, Multi-surface modeling

## Abstract

**Aims:**

To test if multi–surface models can provide a soil-specific prediction of metal mobilization by phytosiderophores (PS) based on the characteristics of individual soils.

**Methods:**

Mechanistic multi-surface chemical equilibrium modeling was applied for obtaining soil-specific predictions of metal and PS speciation upon interaction of the PS 2’-deoxymugineic acid (DMA) with 6 soils differing in availability of Fe and other metals. Results from multi-surface modeling were compared with empirical data from soil interaction experiments.

**Results:**

For soils in which equilibrium was reached during the interaction experiment, multi-surface models could well predict PS equilibrium speciation. However, in uncontaminated calcareous soils, equilibrium was not reached within a week, and experimental and modeled DMA speciation differed considerably. In soils with circum-neutral pH, on which Fe deficiency is likely to occur, no substantial Fe mobilization by DMA was predicted. However, in all but the contaminated soils, Fe mobilization by DMA was observed experimentally. Cu and Ni were the quantitatively most important metals competing with Fe for complexation and mobilization by DMA.

**Conclusion:**

Thermodynamics are unable to explain the role of PS as Fe carrier in calcareous soils, and the kinetic aspects of metal mobilization by PS need to be closer examined in order to understand the mechanisms underlying strategy II Fe acquisition.

**Electronic supplementary material:**

The online version of this article (doi:10.1007/s11104-014-2128-3) contains supplementary material, which is available to authorized users.

## Introduction

Strategy II Fe acquisition, which is employed by graminaceous plants, is characterized by root exudation of a class of chelating agents called phytosiderophores (PS) (Takagi et al. 1984; Takagi 1976), for the purpose of facilitating the transport of soil-Fe towards the root surface (Marschner et al. 1986). Upon exudation, PS diffuse away from the root towards a soil particle, where the PS chelate and mobilize Fe (Kraemer et al. 2006; Lindsay and Schwab 1982). At the root surface the FePS complex is taken up by a high affinity transporter (Römheld and Marschner 1986).

Current research largely focuses on the development of analytical methods to detect and quantify PS in natural matrices (Dell'mour et al. 2012; Koster et al. 2011; Tsednee et al. 2012) and to clear up plant regulation of PS production, exudation and uptake (Daneshbakhsh et al. 2013; Kudo et al. 2013; Lee et al. 2012; Nozoye et al. 2013). The interaction of PS with soil has been addressed in a number of studies, particularly in the late 1980s and early 1990s, but has received relatively little attention and many aspects are still poorly understood. It was discovered that PS do not exclusively bind and mobilize Fe, but also several other trace nutrients, particularly Zn, Cu and Mn (Treeby et al. 1989; Zhang et al. 1991), as well as Ni and Cd (Awad and Römheld 2000). Furthermore, as a result of microbial degradation of the metal-PS complexes, metal mobilization proved to be only temporary (Takagi et al. 1988).

The extent to which binding of other metals may compromise Fe acquisition by soil-grown graminaceous plants has been subject of debate. Based on basic aqueous equilibrium modeling, using a set of soil relevant stability constants (Lindsay 1979), and predicting metal activities in soil solution by means of solubility equilibria with hypothetical soil metal (hydr) oxide phases, Crowley et al. (1987) stated that molar PS concentrations would be required in order to provide plants with sufficient Fe (20 nM) to support plant growth. Römheld (1991) argued that the modeling results by Crowley et al. (1987) were invalid, because in several studies examining metal mobilization by PS from calcareous soils, substantial Fe mobilization had been demonstrated, with Fe accounting for 20 to 40% of the micronutrients mobilized on average (Marschner et al. 1989; Treeby et al. 1989). As main explanatory factors for this discrepancy to modeling results, Römheld suggested a too low value for the reported FePS complexation constant and kinetic aspects playing a role. Von Wiren et al. (2000) showed that under alkaline soil conditions, FePS complexes were stabilized by participation of the deprotonated hydroxyl group on the 3” carbon in the Fe binding (FeOHPS), and established complexation constants for these complexes.

Reichman and Parker (2005) presented an important first step towards a soil specific prediction of PS speciation, using purely empirical models based on linear regression to describe trace metal solubility as a function of soil parameters such as pH, organic matter content and total metal content. Their modeling results identify Cu and Ni and to a lesser extent Zn as potentially important competitors for Fe with regard to coordinative binding to PS under calcareous soil conditions.

Important limitations of the modeling studies so far are the lack of validation with empirical data (Reichman and Parker 2005) and the lack of a mechanistic description of metal speciation in soils; metal availability has been described through an infinite reservoir rather than a soil reactive metal content. As a consequence, metal activities have been fixed, remaining unaffected by PS addition, thereby disregarding potential partial depletion of reactive metal pools.

In the past two decades, great advances have been made in developing mechanistic surface complexation models (SCM) for describing the adsorption of metals to reactive soil constituents (e.g. Fe (hydr) oxides and soil organic matter). By combining these SCM in a multi-surface approach and considering soil properties and the presence of synthetic ligands, soil specific estimations of metal activities and metal speciation can be simulated on a mechanistic basis (Schenkeveld et al. 2010b; Weng et al. 2001).

We hypothesize that by multi–surface modeling, a soil specific prediction of metal mobilization from soils by PS can be made based on the characteristics of individual soils. To test this hypothesis, multi-surface models were composed for a set of actual soils differing in soil properties, in particular metal availability, and addition of the PS 2’-deoxymugineic acid (DMA) to these soils was simulated. The model outcomes were compared with data from metal mobilization experiments using the same soils to verify if equilibrium chemistry can provide a meaningful prediction of DMA solution speciation.

## Materials and methods

### Model description

Metal speciation (Fe, Cu, Ni, Zn, Mn, Al, and Co) was modeled for 6 soils (see materials) using the computer program ECOSAT (Keizer and van Riemsdijk 1994), and addition of DMA to these soil was simulated. Model validation was done by comparing metal activities in the simulated soil systems without DMA addition, with the metal activities determined in CaCl_2_ extracts of the corresponding soils.

Protonation and metal complexation constants for DMA were taken from von Wiren et al. (2000) and Murakami et al. (1989), and were corrected to μ = 0 with the Davies equation (SI-Table 1). Metal speciation in soils was modeled using a multi-surface approach, in which soils are considered a set of independent reactive surfaces (Weng et al. 2001). Adsorption to these surfaces is assumed to be linearly additive, implying that there is no interaction between the surfaces. This multi-surface approach has been successfully applied to predict free metal concentrations in soils (Dijkstra et al. 2004; Weng et al. 2001).

Metal and proton binding to soil organic matter (SOM) and dissolved organic matter (DOM) were described with the Non-Ideal consistent Competitive Adsorption (NICA)-Donnan model (Kinniburgh et al. 1999). The carbon content of SOM and DOM was assumed to be 50%. Humic acid (HA) was used as a model analogue for SOM. The maximum binding capacity (Q_max_) of SOM was assumed to be one third of the binding capacity of HA (i.e. 5.7 mol kg^−1^ eq.) (Weng et al. 2001). DOM was modeled as if it would comprise 30% HA, 30% fulvic acid (FA) and 40% inert material (Weng et al. 2002). Generic NICA-Donnan parameters for metal and proton binding to HA and FA were taken from Milne et al. (2001; 2003).

Adsorption to crystalline Fe (hydr) oxide surfaces was described with the Charge Distribution Multi Site Complexation (CD-MUSIC) model (Hiemstra and Van Riemsdijk 1996; 1999) using goethite as a modeling analogue. Adsorption to amorphous Fe (hydr) oxides was described with the Diffuse Double Layer (DDL) model (Dzombak and Morel 1990) using hydrous ferric oxide (HFO) as a modeling analogue. Both models were parameterized according to Weng et al. (2001). Additionally, constants for Ni and Co adsorption to goethite were fitted based on the data presented by Ponthieu et al. (2006) (SI-Table 2). Specific metal sorption to Al- and Mn (hydr) oxides and clay minerals was not included in this study. Adsorption of DMA ligand and metal-DMA complexes was not included into the models.

The reactive metal contents of the soils were determined with a 0.43 M HNO_3_ extraction (Tipping et al. 2003); the amorphous Fe (hydr) oxide contents were determined with an ammonium oxalate extraction (Schwertmann 1964), and the crystalline Fe (hydr) oxide contents were determined from the difference in extracted Fe between a bicarbonate-dithionite-citrate extraction (Jackson et al. 1986), and an ammonium-oxalate extraction. The soil organic matter content was determined by loss on ignition.

Metal activities in 10 mM CaCl_2_ extracts were calculated from the pH of the extract, the DOM concentration and the total metal concentrations in the extracts (Weng et al. 2002).

Soil conditions were modeled in accordance with the batch interaction experiments: a soil-solution ratio (SSR) of 1 and 10 mM CaCl_2_ as background electrolyte. Fe and Al activity were imposed by the solubility of the respective hydroxide minerals. The solubility product of Fe (OH)_3_ was set to 10^-39.3^ and the solubility product of Al (OH)_3_ was set to 10^-32.34^, corresponding to experimentally observed typical solubilities in soils (Lindsay 1979). Mn activity was constrained by the solubility of MnO (K_s_ = 10^-9.61^).

### Materials

#### Soils

Soils were collected at five sites located in Spain (Santomera and Xeraco) and Austria (Siebenlinden, Redlschlag and Arnoldstein). At all sites, the top soil was sampled (0–20 cm); at the Xeraco site also the soil layer directly underneath was sampled (20–40 cm). All soils have a pH at which Fe deficiency may occur, with exception of the Siebenlinden soil which was included as a reference soil. On the Spanish soils, Fe deficiency in plants was actually observed (Schenkeveld et al. 2008; 2010a). The soils were selected to cover a range of soil properties and metal availabilities. The Santomera, Xeraco and Siebenlinden soil are agricultural soils, the Arnoldstein soil is contaminated (Zn, Pb, Cd) by anthropogenic activity (metal smelter) and the Redlschlag soil is naturally enriched in Ni as a result of serpentinite weathering. Soils were air-dried and sieved over 2 mm. Selected soil parameters were determined and are presented in Table 1.

**Table 1:** Soil characteristics of uncontaminated calcareous soils (Santomera, Xeraco L and Xeraco T), an acidic reference soil (Siebenlinden) and contaminated soils (Redlschlag and Arnoldstein A) used in soil interaction experiments with DMA, and in multi-surface modelling

	pH (CaCl_2_)	SOM content (g kg^−1^)	Clay content (g kg^−1^)	CaCO_3_ content (g kg^−1^)	Crystalline Fe (hydr) oxide content (g kg^−1^)	Amorphous Fe (hydr) oxide content (g kg^−1^)	DTPA-extractable Cu (mg kg^−1^)	Ni (mg kg^−1^)	Zn (mg kg^−1^)	Mn (mg kg^−1^)	Co (mg kg^−1^)	Fe (mg kg^−1^)
Santomera	7.5	15	300	499	15.4	0.9	1.6	0.3	0.5	3.1	0.0	4.9
Xeraco L	7.4	28	430	415	24.3	4.2	3.1	0.4	5.7	14.1	0.1	7.5
Xeraco T	7.2	57	160	147	4.3	4.7	1.4	0.5	6.6	8.9	0.1	75.7
Siebenlinden	4.9	26.5	100	9	12.4	5.4	0.1	0.1	1.3	6.5	0.0	47.3
Redlschlag	6.9	27	190	3	18.3	6.3	0.6	32	0.8	22.8	0.6	13.1
Arnoldstein A	7.2	66	235	323	18.9	7.1	8.3	0.7	122	8.0	0.1	18.0
0.43 M HNO3-extractable						CaCl_2_-extractable						
	Cu	Ni	Zn	Mn	Co	DOC	Cu	Ni	Zn	Mn	Co	
	(mg kg^−1^)	(mg kg^−1^)	(mg kg^−1^)	(mg kg^−1^)	(mg kg^−1^)	mg/l	μM	μM	μM	μM	μM	
Santomera	2.5	6.2	1.3	117	2.2	4.9	0.027	0.17	0.020	0.078	0.014	
Xeraco L	7.6	3.9	17.1	172	2.2	24.4	0.18	0.18	0.015	1.32	0.019	
Xeraco T	4.6	7.5	20.4	148	1.3	97.5	0.24	0.42	0.20	3.26	0.04	
Siebenlinden	1.0	0.4	7.4	118	1.2	44.8	0.041	0.32	1.21	66.5	0.29	
Redlschlag	4.3	182	5.2	168	22	8.3	0.033	4.40	0.20	3.33	0.058	
Arnoldstein A	30.0	7.0	645	480	3.4	78.2	1.04	0.48	2.98	6.88	0.093	

#### DMA solution

Ammonium-DMA salt was synthesized in accordance with Namba et al. (2007). The compound was characterized by LC-ESI-TOF-MS (Agilent Time-of-Flight LC/MS 6220 system) with a specified mass accuracy of ≤ 2 ppm that allows for compound confirmation via sum formula determination. Purity was determined to be higher than 95% by H-NMR. The compound can be readily dissolved in water. Experimental solutions were prepared from analytical grade chemicals and ultra-pure water.

### Experiments

Metal mobilization from soils by DMA was examined in a series of batch interaction experiments. NaN_3_ (2 g L^−1^) was added to the DMA solution as a sterilant to prevent microbial degradation of the DMA ligand. 10 mM CaCl_2_ was used as background electrolyte. Blank treatments (without DMA) were also included. Experiments were carried out in duplicates.

Metal mobilization experiments were carried out in 50 ml polypropylene centrifuge tubes (VWR Eur. Cat. No. 525–0224). A 100 μM DMA solution was added to soil in a SSR of 1 (w/v). The samples were put in an end-over-end shaker rotating at 18 rpm in the dark at 20 °C. Sampling was done destructively after 4, 48 and 96 h for the Redlschlag and Arnoldstein A soil, and after 4, 96 and 168 h for all other soils. Several sampling times were included in order to determine if chemical equilibrium regarding DMA speciation was reached in the course of the experiment. The sampling after 4 h was included, because this time scale is relevant in view of the diurnal release of PS by grasses (Takagi et al. 1984). Samples were centrifuged for 5 min at 4,500 rpm. The pH of the supernatant was measured and the supernatant was filtered over a 0.45 μM cellulose acetate filter (Whatman Aqua 30/0,45 CA). The filtrates were further analyzed as described below.

### LC-MS/MS analysis of total DMA concentration

Separation of DMA was achieved on an Agilent 1200SL HPLC system using a 150×2.1 mm Hypercarb® 3μm porous particle size column via gradient elution chromatography. The eluents comprised Eluent A (98% v/v H_2_O, 1% v/v formic acid, 1% v/v methanol) and Eluent B (98% v/v methanol, 1% v/v formic acid, 1% v/v H_2_O). For gradient elution Eluent A was increased to 20% within 0 min to 6 min and re-equilibrated to starting condition at 6.10 min. The total chromatographic run time was 14 min. The injection volume was 5 μL and the column oven temperature was set to 60 °C. The chromatographic system was combined with an ESI-MS/MS mass selective detector (Agilent 6410 Triple Quadrupole system) for selective quantification of the total DMA ligand concentration. Employing LC-ESI-MS/MS in selected ion monitoring mode with collision induced dissociation (m/z 305.1 -> 186.2) allowed exclusion of co-eluting compounds and reduction of background noise, thereby increasing sensitivity and signal-to-noise ratio. For the purpose of sample stabilization and dissociation of putatively present metal-DMA complexes, all samples were acidified to 1% formic acid in order to obtain the free DMA ligand. Quantification was performed via external calibration (0.1 to 80 μM DMA) with standardization using an in-house synthesized ^13^C_4_-DMA as internal standard (final concentration of 3.33 μM in samples and standards). When necessary, samples were diluted to concentrations within the calibration range. Neither CaCl_2_ nor NaN_3_ in the samples interfered with the measurement (Schindlegger et al. 2014).

### Metal and DOC analysis

Metal concentrations were measured by ICP-MS (Perkin Elmer, ELAN 6100: Al, Cd, Co, Cr, Cu, Ni, Mn, Pb and Zn) and ICP-OES (Optima 5300 DV, Perkin Elmer: Al, Fe, Pb, Zn). Samples were acidified with nitric acid before ICP-analysis. The concentrations of metal-DMA complexes were calculated from the difference in metal concentration between the treatment involving DMA and the corresponding blank treatment. The free DMA ligand concentration was calculated from the difference between the total DMA ligand concentration and the total metal-DMA concentration (i.e. the sum of all metal-DMA concentrations). The presence of free DMA ligand was established by a simple t-test (α = 0.05), comparing both aforementioned concentrations. DOC concentrations were measured with a TOC-5000 (Shimadzu).

## Results

### Soil interaction experiments

**Fig. 1:**
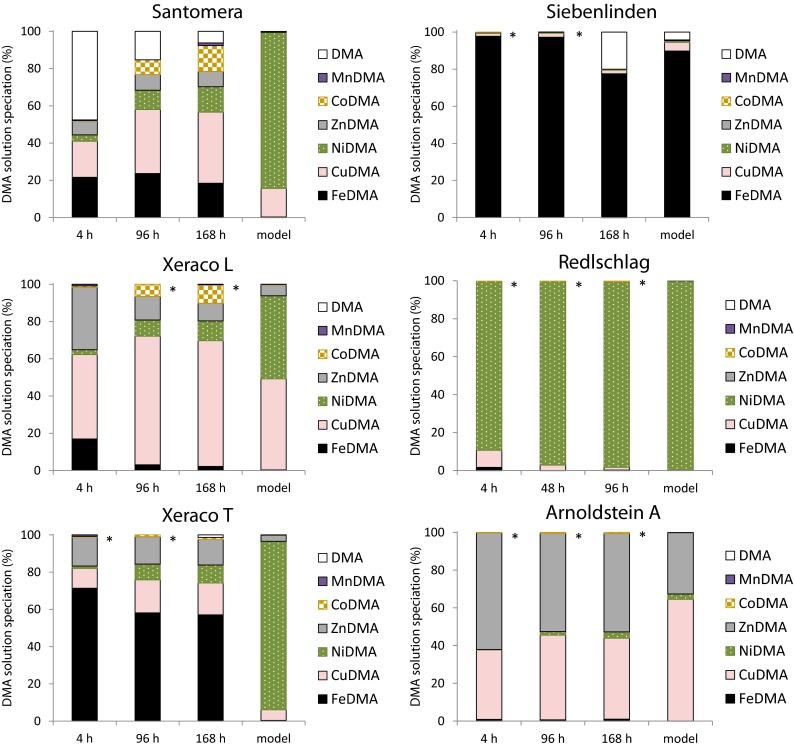
Solution species distribution of DMA as measured after 4, 96/48 and 168/96 h of interaction between various soils and a 100 μM DMA solution, and as predicted by means of equilibrium modeling. The DMA solution contained 10 mM CaCl_2_ as background electrolyte and 2 g l^−1^ NaN_3_ as sterilant. Soil-solution ratio = 1. * No free DMA ligand measured for this time point

Upon interaction with soils, DMA mainly mobilized Fe, Cu, Zn, Ni and Co (Fig. 1). Marginally mobilized were Mn (sub μM range) and Cd (lower nM range; data not shown). No significant mobilization of Al, Pb or Cr was observed. Mobilization of Fe and other metals is discussed in more detail in the following paragraphs. In Fig. 1 the concentrations of the DMA species are normalized to the total amount of DMA in solution – actual concentration data are presented in SI-Table 3. Adsorption of DMA by soil reactive phases will hardly affect the concentration ratios as long as the effect of DMA addition on the free metal concentration remains small, or if the DMA species adsorb to a comparable extent.

A substantial fraction of the DMA ligand and DMA complexes adsorbed onto the soil solid phase; adsorption, calculated as the difference between the total DMA solution concentration at t=0 (100 μM) and at t = t, accounted for 25 to 62 % of the DMA ligand added (SI-Table 3). Metal mobilization was strongly soil dependent: from the Xeraco T and Siebenlinden soil mainly (>50%) Fe was mobilized, from the Redlschlag soil mainly Ni, from the Xeraco L soil mainly Cu, and from the Arnoldstein A soil mainly Zn and Cu. For the Santomera soil, Cu was mobilized to the largest extent, but Fe, Ni, Co and Zn complexes all contributed substantially to the overall DMA speciation.

### Fe mobilization

FeDMA was the dominant DMA species upon interaction of DMA with Siebenlinden soil (>85%) and Xeraco T soil (>55%). Fe was also mobilized, yet to a lesser extent, from Santomera soil (18–24%) and Xeraco L soil (2–17%). For the contaminated Redlschlag and Arnoldstein A soil, Fe mobilization was negligible with FeDMA accounting for less than 2% of the DMA solution speciation. Despite the fact that Siebenlinden and Xeraco T soil had the highest DTPA-extractable Fe contents (Table 1), the extent to which Fe is mobilized from soils by DMA cannot be simply linearly related to this Fe availability parameter. Availability of other metals that compete for binding by DMA affect Fe mobilization as long as the amount of DMA ligand added is limiting. This is clearly demonstrated for the Redlschlag and Arnoldstein A soil, which have a higher DTPA-extractable Fe content than the Santomera and Xeraco L soil, yet much less Fe is mobilized from the former soils, because their availability contents of other metals is also much higher (Table 1).

### Mobilization of other metals

CuDMA contributed to the DMA solution speciation in all soils, to some extent. After 4 h the CuDMA concentration ranged from 0.9 μM in Siebenlinden soil to 27.3 μM in Arnoldstein A soil. The Cu concentration was a factor 23 to 280 higher than in corresponding 10 mM CaCl_2_ extracts (Table 1). In the uncontaminated Spanish calcareous soils, Cu proved to be the quantitatively most important competing metal for DMA binding to Fe. Ni was mobilized from all soils except Siebenlinden, making a relatively small contribution to the overall DMA solution speciation in the Arnoldstein A soil (0.2 μM after 4 h), and completely dominating it in the Redlschlag soil (45.0 μM after 4 h). After 4 h of interaction, Ni mobilization by 100 μM DMA was up to a factor 11 higher than in 10 mM CaCl_2_ extracts. In all soils where Ni was mobilized by DMA the relative contribution of NiDMA to DMA speciation increased over time. Zn mobilization occurred in all soils except Siebenlinden and Redlschlag; after 4 h it ranged from 2.9 μM in Santomera soil to 46 μM in Arnoldstein A soil. Zn mobilization was a factor 16 to 1,100 higher than in the corresponding 10 mM CalCl_2_ extracts. CoDMA only substantially contributed to overall DMA solution speciation (>2%) in the calcareous clay soils Santomera and Xeraco L. Also in these soils, CoDMA concentrations were still negligible (<0.4%) after 4 h, but concentrations gradually increased up to 5–7 μM after 168 h. Despite the very high reactive Pb content of the Arnoldstein A soil (3.6 g kg^−1^), no Pb mobilization by DMA was found.

### Free DMA ligand

Upon interaction of DMA with Santomera soil, substantial free DMA ligand concentrations were found for every time point. After 4 h, free DMA was even the quantitatively most important DMA species (48%). As a result of gradual metal mobilization, the free DMA concentration decreased over time (Fig. 1, SI-Table 3). The fact that in no other soil such slow metal dissolution rates were observed is presumably related to the fact that Santomera soil has the lowest overall metal availability (Table 1 – DTPA extractable metals).

For the Xeraco T and Xeraco L soils, already after 0.25 h no statistically significant free DMA ligand concentrations were present (p > 0.05; data not shown). For the Siebenlinden soil total DMA concentrations were only measured after 0.25 h (data not shown) and 168 h. For both time points statistically significant free DMA concentrations were calculated (respectively 6.0 and 10.2 μM; p < 0.05). For the DMA solution speciation presented in Fig. 1, data on the free ligand is missing for t = 4 h and t = 96 h.

No analysis of total DMA concentration was carried out for the Redlschlag and Arnoldstein A soil, and hence no free DMA ligand concentrations could be calculated. However, metal availability in these soils is very high, and free DMA ligand concentrations at any sample moment were assumed negligible.

### Time dependence of DMA speciation

Comparison of the DMA species distributions for the individual soils between the different sampling moments indicate that only for the Siebenlinden soil steady state was reached within 4 h. For the Redlschlag and Arnoldstein A soil, changes in species distribution were limited between 4 and 48 h, and after 96 h steady state seems to be approached. For the Santomera, Xeraco L and Xeraco T soils, steady state clearly had not yet been reached after 4 h. Between 96 and 168 h, the CoDMA, NiDMA and CuDMA fractions in Santomera soil still increased, while the FeDMA and free DMA ligand fractions still decreased. For Xeraco L soil, CoDMA and NiDMA fractions still increased, while the ZnDMA fraction decreased. For Xeraco T, soil changes in species distribution are less pronounced, yet the NiDMA fraction still increased after 96 h. For Santomera and Xeraco L soil, and possibly also for Xeraco T soil, steady state was not reached within the time-span of the experiment.

## Modeling results

### Metal speciation without DMA

Predicted metal activities and metal speciation in the modeled soils are presented in Table 2. For metals of which the activity was imposed by a solid phase, or that were not predicted to be substantially complexed by DMA (<0.5% of total DMA in all soils), only the activity is presented. Without DMA addition log(Cu^2+^) ranges from −12.4 to –9.7; log(Ni^2+^) from −7.6 to –4.5; log(Zn^2+^) from −9.1 to –4.2; log(Fe^3+^) from −19.8 to –12.0; log(Al^3+^) from −12.8 to –5.0; log(Co^2+^) from −7.9 to –5.3; and log(Mn^2+^) from −4.2 to –3.2. Fe^3+^ and Al^3+^ activities are highest in the Siebenlinden soil, due to the strong dependency of the solubility of hydroxide minerals on pH, and the relatively low pH of this soil. Ni and Co activities are highest in the serpentine Redlschlag soil. Zn and Cu activities are highest in the anthropogenically contaminated Arnoldstein A smelter soil.

**Table 2:** metal speciation in 10 mM CaCl_2_ soil suspensions in absence and presence of 100 μM DMA as predicted through multi-surface modeling

Soil	log (Cu^2+^)	Cu-OM	Cu-Feox-cr	Cu-Feox-am	CuDMA	log (Ni^2+^)	Ni-OM	Ni-Feox-cr	Ni-Feox-am
Santomera	−12.1	92.7%	7.2%	0.1%	-	−7.2	9.5%	87.3%	3.2%
	−12.6	58.8%	1.9%	0.1%	39.2%	−8.0	3.3%	16.8%	0.6%
Xeraco L	−11.4	88.0%	11.5%	0.5%	-	−7.6	17.9%	74.5%	7.6%
	−12.0	55.9%	3.1%	0.2%	40.8%	−8.1	8.5%	22.2%	2.3%
Xeraco T	−12.4	99.8%	0.1%	0.0%	-	−6.7	60.7%	19.9%	19.4%
	−12.5	92.0%	0.1%	0.0%	7.9%	−7.4	21.5%	4.0%	3.9%
Siebenlinden	−10.1	99.9%	0.0%	0.0%	-	−7.1	94.4%	0.0%	5.5%
	−10.4	70.2%	0.0%	0.0%	29.7%	−7.2	83.1%	0.0%	4.5%
Redlschlag	−11.1	96.9%	2.8%	0.3%	-	−4.5	23.3%	37.2%	39.6%
	−11.1	96.6%	2.8%	0.3%	0.3%	−4.5	22.5%	36.3%	38.0%
Arnoldstein A	−9.7	69.5%	29.2%	1.3%	-	−7.2	33.3%	62.2%	4.4%
	−9.8	63.1%	22.3%	1.0%	13.7%	−7.3	31.7%	57.8%	4.1%
Soil	log (Zn^2+^)	Zn-OM	Zn-Feox-cr	Zn-Feox-am	ZnDMA	log (Fe^3+^)	log (Al^3+^)	log (Co^2+^)	log (Mn^2+^)
Santomera	−9.1	97.1%	2.2%	0.7%	-	−19.8	−12.8	−7.8	−3.9
	−9.1	96.8%	2.1%	0.7%	0.4%	−19.8	−12.8	−7.9	−3.9
Xeraco L	−7.0	53.6%	23.1%	23.3%	-	−19.5	−12.5	−7.9	−3.9
	−7.0	53.0%	22.2%	22.5%	2.3%	−19.5	−12.5	−7.9	−3.9
Xeraco T	−6.9	80.4%	2.3%	17.3%	-	−18.9	−11.9	−7.2	−4.2
	−6.9	79.6%	2.3%	17.1%	1.1%	−18.9	−11.9	−7.2	−4.2
Siebenlinden	−5.6	85.9%	0.4%	13.7%	-	−12.0	−5.0	−5.8	−3.6
	−5.6	85.6%	0.4%	13.6%	0.4%	−12.0	−5.0	−5.8	−3.6
Redlschlag	−7.2	84.1%	6.6%	9.3%	-	−18.0	−11.0	−5.3	−3.7
	−7.2	83.9%	6.6%	9.5%	0.0%	−18.0	−11.0	−5.3	−3.7
Arnoldstein A	−4.2	18.3%	30.3%	51.4%	-	−18.9	−11.9	−7.1	−3.2
	−4.2	18.2%	30.2%	51.2%	0.3%	−18.9	−11.9	−7.1	−3.2

In absence of DMA, 70–100 % of soil-Cu is bound to SOM, reflecting the high affinity of the organic matter functional groups for Cu. In the Arnoldstein A soil, which has both the highest Cu activity and the highest reactive Cu-content, approximately 30% of the Cu is bound to Fe (hydr) oxide minerals, mainly to the crystalline phases.

The distribution of Ni over the soil reactive surfaces is more soil dependent. Ni is mainly bound to SOM (>60%) in the Xeraco T and the Siebenlinden soil, which are both sandy soils and have the lowest crystalline Fe (hydr) oxide content. In the clay soils (Santomera, Xeraco L and Arnoldstein A), Ni is mainly bound to crystalline Fe (hydr) oxides (>60%). In the Redlschlag soil, which has the highest reactive Ni content, Ni is largely bound to Fe (hydr) oxides (ca. 75%), approximately equally distributed over amorphous and crystalline phases.

With exception of the Arnoldstein A soil, most soil-Zn is bound to SOM (>50%). In the Arnoldstein A soil, which has the highest reactive Zn content by large, most Zn is bound to Fe (hydr) oxides (amorphous: ca. 50%; crystalline: ca. 30%).

### Metal speciation with DMA addition

Only the activities of Cu^2+^ (up to 0.6 log units) and Ni^2+^ (up to 0.8 log units) are substantially (>0.1 log unit) affected by the simulated DMA addition; the activities of Fe and Al were imposed by a mineral hydroxide phase, formation of CoDMA complexes was not included in the modeling because no complexation constant has been reported, and the formation of Mn(II)DMA and ZnDMA complexes is negligible relative to the total reactive Mn and Zn pools in the soils. Therefore simulated DMA addition hardly leads to changes in the distribution of Zn over the reactive surfaces, despite the predicted Zn complexation by DMA in certain soils (Fig. 1; Table 2). In the Santomera, Xeraco L and Xeraco T soil, ca. 30 to 40% of the reactive Cu^2+^ is predicted to become complexed by DMA; for Ni^2+^ it is even ca. 65 to 80%; this indicates a substantial depletion of the reactive metal pools. Both for Cu and Ni, the model simulations predict a larger relative decrease in Fe (hydr) oxides bound metal than in SOM bound metal.

### DMA speciation

The DMA speciation predicted from the multi-surface models is presented in Fig. 1 (see SI-Table 3 for the exact numbers). Fe is only predicted to be substantially complexed by DMA (>0.5%) in the Siebenlinden soil (ca. 90%). This soil was included as a reference and has a relatively low pH (4.9), making Fe deficiency in plants grown on this soil improbable. An important result of these model calculations is that 100 μM DMA has little effect on the equilibrium solubility of Fe in calcareous soils. Although the stability constants for the FeDMA complex are higher than for the complexes of competing metals (SI-Table 1; Murakami et al. 1989; von Wirén et al. 2000), Fe is outcompeted by other metals due to its very low activity in calcareous soil (Table 2). The predicted lack of FeDMA formation in calcareous soil is consistent with previous modeling studies (Crowley et al. 1987; Reichman and Parker 2005). Hence it can be concluded that, from a thermodynamic perspective, the phytosiderophore DMA is not an Fe specific chelating agent in a soil environment and equilibrium chemistry is unable to explain its role as an Fe carrier.

CuDMA and NiDMA are predicted as the quantitatively most important species under equilibrium conditions in soils with a circum-neutral pH (Fig. 1, Table 1). In the Santomera, Xeraco T and Redlschlag soils, NiDMA is predominant (>80%); in the Xeraco L soil, approximately equal concentrations of NiDMA and CuDMA are predicted (40 – 50%); and in the Arnoldstein A soil, CuDMA is predicted as the main DMA species (>60%). ZnDMA is predicted to be a quantitatively important species (>30%) in the Arnoldstein A soil and of quantitatively lesser importance (3 – 6%) in the Xeraco T and L soils. MnDMA is quantitatively not a relevant species in any of the soils (<0.5%), despite the predicted high Mn activities. In the Siebenlinden soil, a fraction of the DMA (ca. 4%) is predicted to be present as mere ligand.

The predominance of the NiDMA species in the Redlschlag soil is caused by the high natural Ni abundance in this soil. Despite a 20-fold higher reactive soil content of Zn than of Cu in the Arnoldstein A soil, complexation of Cu can still effectively compete with Zn, due to the substantially higher logK value for the CuDMA complex (19.98) than for the ZnDMA complex (14.12). In the uncontaminated calcareous soils, Ni and Cu are the main competitors for binding by the DMA ligand. The reactive Ni and Cu soil contents are in the same order of magnitude (Table 1); Ni is bond less strongly by both soil and DMA ligand. If the Ni activity is 4 or more orders of magnitude higher than the Cu activity (after reaction with the DMA ligand) (Table 2), NiDMA will dominate DMA speciation; if the difference is smaller or Cu activity is higher, CuDMA will dominate.

The free DMA ligand in the Siebenlinden soil arises from the fact that at lower soil pH, protons compete more effectively with metals for binding to the functional groups of the DMA ligand. The crystallinity of the Fe (hydr) oxide phase also plays an important role in how large the actual free DMA concentration will be (see model verification).

## Discussion

### Verification of the multi-surface model metal activities by means of CaCl_2_ extracts

The multi-surface models were validated by comparing the calculated activities from the simulations without DMA addition with the activities calculated from measured metal and DOC concentrations in CaCl_2_ extracts. A comparison of the data is presented in SI-Fig. 1 included in the supporting information.

For Cu, the activities calculated with the multi-surface models and from the CaCl_2_ extracts compare very well – for none of the soils a deviation larger than 0.7 log units was found. For Ni, a deviation larger than 0.4 log units was only found for the Redlschlag soil (1.2 log units); the difference is presumably caused by disregarding the Ni containing serpentinite mineral phase in the multi-surface model for this soil. For predicting the DMA speciation in the Redlschlag soil, this difference in activity has little effects; even with the activity determined from the CaCl_2_ extract, DMA would nearly exclusively bind Ni.

For Zn, the multi-surface activity predictions are only in good agreement with the activities determined from the CaCl_2_ extract for the Santomera, Redlschlag and Siebenlinden soil (deviation < 0.7 log unit); for the Arnoldstein A, Xeraco L and Xeraco T soil, deviations are considerable (1.9 – 4.0 log units), and the multi-surface activity is consistently higher. The latter soils have the highest reactive Zn contents and are all calcareous soils; disregarding Zn precipitates in the multi-surface models could explain the differences in activity. Allowing for precipitation of ZnCO_3_ (pK_sol_ = 10.24; P_CO2_ = 2.88 10^−4^ bar) or ZnO (pK_sol_ = 16.84) in the multi-surface models did not affect Zn activity. With regard to DMA speciation, exactly for these three soils the multi-surface models predict that a certain fraction of DMA would bind Zn. A substantially lower Zn activity would also considerably lower the fraction of DMA binding Zn.

For Mn, the multi-surface model consistently predicts a (substantially) higher activity (0.9 – 3.5 log units) than the activity calculated from the CaCl_2_ extract. By disregarding the redox chemistry of Mn in the multi-surface model and assuming all Mn is present as Mn (II), an overestimation of the Mn^2+^ activity is certain, especially at the relatively high pH of calcareous soils. However, results from the multi-surface models demonstrate that despite the overestimation of the Mn activity, the Mn(II)DMA complex is of marginal importance at most with regard to the overall DMA speciation (>0.33%).

For Co, the activities calculated with the multi-surface models and from the CaCl_2_ extracts were in reasonable agreement; a deviation larger than 1.0 log units was only found for the Redlschlag soil (2.2 log units); possibly the difference is also related to disregarding the Co included in the serpentinite mineral phase in the multi-surface model for this soil.

Fe activities in equilibrium with Fe (hydr) oxide are extremely low, except in very acidic or alkaline environments. Therefore, analytical limitations do not allow precise determination of Fe activity in extracts. Furthermore, the choice of 39.3 for pK_sol_ for Fe (hydr) oxide minerals represents an average for soils (Lindsay 1979), but may be inaccurate for some soils under investigation. For example, in calcareous soils with a low SOM content and a high ratio of crystalline to amorphous Fe (hydr) oxides (e.g. Santomera soil), the solubility product is likely to be overestimated. A sensitivity analysis was carried to examine the effect of the pK_sol_ value of soil Fe (hydr) oxides on DMA speciation. This was done for the Santomera soil (highest ratio of crystalline to amorphous Fe (hydr) oxides), Xeraco T soil (lowest ratio of crystalline to amorphous Fe (hydr) oxides) and Siebenlinden soil (highest Fe complexation by DMA). Results are presented as supporting information (SI-Table 4) and indicate that for the calcareous soils, Fe complexation by DMA remains marginal (>2.5%) regardless of the Fe (hydr) oxide solubility. For the Siebenlinden soil, a decreases in Fe (hydr) oxide solubility would largely decrease the DMA fraction binding Fe, from ca. 90% for soil-Fe to only 3% for goethite, mainly at the benefit of free DMA ligand (up to 75%).

### Comparison modeling results with experimental results – synthesis

The modeling outcomes predict concentration ratios under chemical equilibrium conditions. For comparing the modeling and the experimental results it is therefore important to distinguish between soils for which (approximate) equilibrium was reached during the interaction experiment and soils for which this was (presumably) not yet the case. Soils for which metal concentrations reached a steady state, chemical equilibrium is assumed.

Soils for which equilibrium was reached are Siebenlinden, Redschlag and Arnoldstein A soil. For these soils the model predictions are good: multi-surface equilibrium modeling could predict the dominant metals mobilized by DMA. In case of Siebenlinden and Redlschlag there was a single dominant species (FeDMA and NiDMA respectively); for the Arnoldstein A soil, there were two dominant species, namely CuDMA and ZnDMA.

For the Siebenlinden soil the predicted free DMA ligand concentration somewhat underestimates the experimental concentration measured after 168. This might be because the actual solubility of the Fe (hydr) oxide phase of the soil was overestimated in the model (K_s_ = 10^-39.3^), or because of an accumulation of errors in the calculated free DMA ligand concentration. The difference in ZnDMA to CuDMA ratio in the Arnoldstein A soil between the model prediction and experimental data may be caused by inaccuracies in the predicted metal activities or uncertainty in the available complexation constants for the ZnDMA and CuDMA complexes.

For the soils for which equilibrium was not reached within the 1-week timespan of the experiment, the model calculations were much less successful in predicting metal mobilization. A sensitivity analysis in which the K_C_ values of the DMA complexes were varied up to two log units (SI-Table 5) confirmed that it was not possible to obtain good fits between the 168 h experimental data and the model results with one consistent set of K_c_ values for all three soils. In soils that are characterized by slow reaction rates, the development of metal concentrations is consistent with a drift towards the model-predicted equilibrium state. For all three soils, the predicted equilibrium NiDMA fraction was strongly overestimated compared to the experimental data. However, in all cases the NiDMA fraction was still gradually increasing from 96 to 168h (Fig. 1). The slow complexation kinetics of Ni in comparison to other divalent metals might play an important role in this respect. These slow kinetics are related to a slow water exchange rate in the primary hydration shell of Ni. ZnDMA, CuDMA and FeDMA concentrations were in all cases underestimated by the models, yet the fractions of these DMA species were still decreasing from 96 and 168h with exception of the CuDMA fraction in Santomera soil. The increasing CuDMA fraction in the Santomera soil is presumably related to the fact that there was still substantial free DMA ligand present in solution, so the total metal-DMA fraction is still further increasing. The lack of a stability constant for the CoDMA complex proved an important limitation for the model predictions, particularly for the Santomera and Xeraco L soils from which substantial mobilization of Co by DMA took place.

It is unclear how the uptake of metals other than Fe would be affected by complexation by DMA. On the one hand, metal activities decrease as a result of complexation (Table 2), which is unfavorable in accordance with the free ion activity model. On the other hand, mobilization by DMA may increase trace metal uptake, since this is often diffusion limited (Degryse et al. 2008).

Our results demonstrate that in principal multi-surface complexation modeling can offer a good, soil specific prediction of the DMA solution speciation upon interaction of DMA with soil (Fig. 1 - Siebenlinden, Redlschlag and Arnoldstein A). However, upon interaction with uncontaminated calcareous soils (Fig. 1 - Santomera and Xeraco T and L), the time required to reach equilibrium can be so long that the results from equilibrium modeling do not correspond with measured metal mobilization data. The time needed for equilibration greatly exceeds relevant timespans in view of Strategy II Fe acquisition with its characteristic daily pulse releases of PS (Oburger et al. 2014 (accepted); Takagi et al. 1984). For this reason, the applicability of thermodynamic modeling as a predictive tool for metal mobilization by PS is limited, particularly for the type of soils for which it would be most relevant: uncontaminated calcareous agricultural soils.

The slow reaction kinetics involved with metal mobilization by PS from soils help to explain the discrepancy between model predictions (Crowley et al. 1987; Reichman and Parker 2005) and experimental observations (Römheld 1991) in previous studies. Although equilibrium models predict otherwise, PS do mobilize Fe from uncontaminated calcareous soils. To better understand the dynamics and underlying mechanisms involved, we suggest further studies should focus on the kinetic rather than on thermodynamic aspects of metal mobilization by PS.

## Electronic supplementary material

Below is the link to the electronic supplementary material.

ESM 1 (DOCX 108 kb)
