# Supplementary material for: Metal mobilization from soils by phytosiderophores – experiment and equilibrium modeling
Source: Plant Soil. 2014 Jun 4;383(1):59–71. doi: 10.1007/s11104-014-2128-3 (PMC4372826; doi:10.1007/s11104-014-2128-3)
Supplement: Supplementary file 1 — (DOCX 108 kb) [file 11104_2014_2128_MOESM1_ESM.docx]

SI-Table 1: DMA stability constants

| Species | logK | Comp 1 | Comp 2 | Comp 3 | Reference |
| --- | --- | --- | --- | --- | --- |
| HDMA^2-^ | 10.64 | 1 DMA^3-^ | 1 H^+^ |  | Murakami 1989 |
| H_2_DMA^-^ | 19.32 | 1 DMA^3-^ | 2 H^+^ |  | Murakami 1989 |
| H_3_DMA | 22.72 | 1 DMA^3-^ | 3 H^+^ |  | Murakami 1989 |
| H_4_DMA^+^ | 25.46 | 1 DMA^3-^ | 4 H^+^ |  | Von Wiren 2000 |
| H_5_DMA^2+^ | 27.60 | 1 DMA^3-^ | 5 H^+^ |  | Von Wiren 2000 |
| FeDMA | 20.30 | 1 DMA^3-^ | 1 Fe^3+^ |  | Murakami 1989 |
| FeOHDMA^-^ | 17.96 | 1 DMA^3-^ | 1 Fe^3+^ | -1 H^+^ | Murakami 1989 |
| CuDMA^-^ | 19.98 | 1 DMA^3-^ | 1 Cu^2+^ |  | Murakami 1989 |
| CaDMA^-^ | 4.62 | 1 DMA^3-^ | 1 Ca^2+^ |  | Murakami 1989 |
| ZnDMA^-^ | 14.12 | 1 DMA^3-^ | 1 Zn^2+^ |  | Murakami 1989 |
| Mn(II)DMA^-^ | 9.57 | 1 DMA^3-^ | 1 Mn^2+^ |  | Murakami 1989 |
| Fe(II)DMA^-^ | 11.73 | 1 DMA^3-^ | 1 Fe^2+^ |  | Murakami 1989 |
| NiDMA^-^ | 16.06 | 1 DMA^3-^ | 1 Ni^2+^ |  | Murakami 1989 |

SI-Table 2: Parameters for the CD-MUSIC model for Ni and Co binding to goethite

|  | **log K** | **coeff 0-plane** | **coeff 1-plane** |
| --- | --- | --- | --- |
| **FeOH-Ni** | 6.9 | 0.83 | 1.17 |
| **FeOH-NiOH** | -1.6 | 0.83 | 0.17 |
| **FeOH-Nih** | 5.85 | 0.83 | 1.17 |
| **FeOH-NiOHh** | 0.7 | 0.83 | 0.17 |
|  |  |  |  |
| **FeOH-Co** | 7 | 0.83 | 1.17 |
| **FeOH-CoOH** | -1.3 | 0.83 | 0.17 |
| **FeOH-Coh** | 5.95 | 0.83 | 1.17 |
| **FeOH-CoOHh** | 0.9 | 0.83 | 0.17 |

|  |  | **FeDMA** | | **CuDMA** | | **NiDMA** | | **ZnDMA** | | **CoDMA** | | **MnDMA** | | **DMA** | |
| --- | --- | --- | --- | --- | --- | --- | --- | --- | --- | --- | --- | --- | --- | --- | --- |
|  |  | **µM** | **SD** | **µM** | **SD** | **µM** | **SD** | **µM** | **SD** | **µM** | **SD** | **µM** | **SD** | **µM** | **SD** |
| **Santomera** | 4 | 8.4 | 0.4 | 7.5 | 0.1 | 1.3 | 0.0 | 2.9 | 0.0 | 0.1 | 0.0 | 0.1 | 0.0 | 18.4 | 0.6 |
|  | 96 | 11.2 | 0.7 | 16.2 | 0.0 | 4.9 | 0.0 | 4.0 | 0.0 | 3.7 | 0.0 | 0.0 | 0.1 | 7.2 | 1.2 |
|  | 168 | 8.7 | 0.7 | 18.0 | 0.5 | 6.4 | 0.0 | 3.9 | 0.1 | 6.5 | 0.1 | 0.7 | 0.1 | 2.9 | 1.1 |
|  | model | 0.3 |  | 15.4 |  | 83.8 |  | 0.1 |  | 0.0 |  | 0.3 |  | 0.0 |  |
| **Xeraco L** | 4 | 8.3 | 0.6 | 22.4 | 0.6 | 1.3 | 0.0 | 16.6 | 0.1 | 0.3 | 0.0 | 0.4 | 0.1 | b.d. |  |
|  | 96 | 1.7 | 0.8 | 38.7 | 0.6 | 4.9 | 0.0 | 7.1 | 0.2 | 3.7 | 0.1 | 0.0 | 0.4 | n.d. |  |
|  | 168 | 1.3 | 0.4 | 38.8 | 0.9 | 6.1 | 0.0 | 5.5 | 0.3 | 5.7 | 0.0 | 0.2 | 0.1 | n.d. |  |
|  | model | 0.3 |  | 48.9 |  | 44.6 |  | 5.9 |  | 0.0 |  | 0.2 |  | 0.0 |  |
| **Xeraco T** | 4 | 51.7 | 0.0 | 7.8 | 0.2 | 0.9 | 0.0 | 11.3 | 0.1 | 0.2 | 0.0 | 0.6 | 0.0 | n.d |  |
|  | 96 | 39.4 | 0.3 | 12.1 | 0.3 | 5.7 | 0.2 | 9.9 | 0.2 | 0.7 | 0.0 | 0.0 | 0.0 | n.d. |  |
|  | 168 | 39.5 | 0.3 | 11.8 | 0.1 | 6.7 | 0.1 | 9.6 | 0.0 | 0.7 | 0.0 | 0.0 | 0.0 | 0.9 | 0.4 |
|  | model | 0.4 |  | 5.7 |  | 90.5 |  | 3.3 |  | 0.0 |  | 0.1 |  | 0.0 |  |
| **Siebenlinden** | 4 | 50.2 | 1.1 | 0.9 | 0.0 | 0.1 | 0.0 | 0.1 | 0.0 | 0.1 | 0.0 | 0.0 | 0.0 | n.d |  |
|  | 96 | 43.6 | 0.6 | 1.0 | 0.0 | 0.2 | 0.0 | 0.1 | 0.1 | 0.0 | 0.0 | 0.0 | 0.0 | n.d. |  |
|  | 168 | 39.6 | 1.0 | 1.0 | 0.0 | 0.2 | 0.0 | 0.0 | 0.0 | 0.0 | 0.0 | 0.0 | 0.0 | 10.2 | 1.6 |
|  | model | 89.8 |  | 4.7 |  | 0.8 |  | 0.4 |  | 0.0 |  | 0.0 |  | 4.2 |  |
| **Redlschlag** | 4 | 0.8 | 0.1 | 4.6 | 0.1 | 45.0 | 0.2 | 0.0 | 0.0 | 0.1 | 0.0 | 0.0 | 0.0 | n.d. |  |
|  | 48 | 0.0 | 0.4 | 1.5 | 0.0 | 51.7 | 0.1 | 0.0 | 0.0 | 0.1 | 0.0 | 0.0 | 0.0 | n.d. |  |
|  | 96 | 0.1 | 0.0 | 0.8 | 0.0 | 52.3 | 0.2 | 0.0 | 0.0 | 0.2 | 0.0 | 0.0 | 0.0 | n.d. |  |
|  | model | 0.0 |  | 0.2 |  | 99.8 |  | 0.0 |  | 0.0 |  | 0.0 |  | 0.0 |  |
| **Arnoldstein A** | 4 | 0.7 | 0.4 | 27.3 | 0.0 | 0.2 | 0.0 | 46.0 | 0.1 | 0.1 | 0.0 | 0.0 | 0.0 | n.d. |  |
|  | 48 | 0.5 | 0.2 | 31.8 | 0.0 | 1.3 | 0.0 | 36.9 | 0.1 | 0.3 | 0.0 | 0.0 | 0.0 | n.d. |  |
|  | 96 | 0.7 | 0.3 | 30.6 | 0.1 | 2.4 | 0.0 | 37.1 | 0.4 | 0.4 | 0.0 | 0.0 | 0.0 | n.d. |  |
|  | model | 0.0 |  | 64.6 |  | 2.8 |  | 32.5 |  | 0.0 |  | 0.0 |  | 0.0 |  |

SI-Table 3: DMA species concentrations as measured after 4, 96/48 and 168/96 hours of interaction between various soils and a 100 µM DMA solution, and as predicted by means of equilibrium modeling. The DMA solution contained 10 mM CaCl_2_ as background electrolyte and 2 g l^-1^ NaN_3_ as sterilant. Soil-solution ratio = 1; n=2.

n.d.: not determined; b.d.: below level of determination

SI-Table 4: Sensitivity analysis on the effect of solubility of the soil Fe(hydr)oxide phase on DMA speciation. The solubility of ferrihydrite and goethite were taken as extreme cases. Predicted DMA solution speciation (percentages) upon interaction of 100 µM DMA with soils.

| **Soil** | **log Ks (Fe(OH)_3_** | **FeDMA**  **(%)** | **CuDMA**  **(%)** | **NiDMA**  **(%)** | **ZnDMA**  **(%)** | **MnDMA**  **(%)** | **DMA**  **(%)** |
| --- | --- | --- | --- | --- | --- | --- | --- |
| **Santomera** | 38.46 | 1.9 | 15.9 | 81.7 | 0.08 | 0.29 | 0.01 |
|  | 39 | 0.62 | 15.7 | 83.3 | 0.08 | 0.32 | 0.02 |
|  | 39.3 | 0.32 | 15.4 | 83.8 | 0.07 | 0.33 | 0.02 |
|  | 40 | 0.07 | 15.0 | 84.5 | 0.07 | 0.34 | 0.02 |
|  | 42.02 | 0.00 | 14.3 | 85.3 | 0.06 | 0.35 | 0.02 |
| **Xeraco T** | 38.46 | 2.3 | 6.3 | 88.2 | 3.2 | 0.04 | 0.02 |
|  | 39 | 0.73 | 5.9 | 90.0 | 3.3 | 0.05 | 0.02 |
|  | 39.3 | 0.38 | 5.7 | 90.5 | 3.3 | 0.05 | 0.02 |
|  | 40 | 0.08 | 5.3 | 91.3 | 3.2 | 0.05 | 0.02 |
|  | 42.02 | 0.00 | 4.7 | 92.2 | 3.1 | 0.05 | 0.02 |
| **Siebenlinden** | 38.46 | 97.6 | 1.5 | 0.17 | 0.07 | 0.00 | 0.66 |
|  | 39 | 93.7 | 3.3 | 0.49 | 0.24 | 0.00 | 2.2 |
|  | 39.3 | 89.8 | 4.7 | 0.84 | 0.44 | 0.00 | 4.2 |
|  | 40 | 71.5 | 7.8 | 2.2 | 1.7 | 0.00 | 16.8 |
|  | 42.02 | 3.1 | 10.6 | 4.1 | 6.7 | 0.02 | 75.5 |

**SI-Table 5: Sensitivity analysis on logKc values**

|  | **Log Kc** |  |  |  |  |  |  |  |  |  |  |  |
| --- | --- | --- | --- | --- | --- | --- | --- | --- | --- | --- | --- | --- |
|  | **FeOHDMA** | **CuDMA** | **ZnDMA** | **NiDMA** |  | **FeDMA** | **CuDMA** | **NiDMA** | **ZnDMA** | **CoDMA** | **MnDMA** | **Free DMA** |
| **Experimental  after 168 hours** |  |  |  |  |  | *18,4%* | *38,2%* | *13,6%* | *8,3%* | *13,8%* | *1,5%* | *6,2%* |
| **Unchanged** | 17,96 | 19,98 | 14,12 | 16,06 |  | 0,3% | 15,4% | 83,7% | 0,1% |  | 0,3% |  |
|  |  |  |  |  |  |  |  |  |  |  |  |  |
| **Change in Log Kc** |  |  |  |  |  |  |  |  |  |  |  |  |
| **Cu +1** | 17,96 | 20,98 | 14,12 | 16,06 |  | 0,2% | 24,6% | 74,9% | 0,0% |  | 0,2% |  |
| **Fe +1** | 18,96 | 19,98 | 14,12 | 16,06 |  | 2,9% | 14,8% | 81,9% | 0,1% |  | 0,3% |  |
| **Zn +1** | 17,96 | 19,98 | 15,12 | 16,06 |  | 0,3% | 15,3% | 83,3% | 0,6% |  | 0,3% |  |
| **Ni -1** | 17,96 | 19,98 | 14,12 | 15,06 |  | 1,7% | 24,0% | 72,0% | 0,4% |  | 1,8% |  |
|  |  |  |  |  |  |  |  |  |  |  |  |  |
| **Ni -1 & Cu +1** | 17,96 | 20,98 | 14,12 | 15,06 |  | 1,3% | 31,0% | 65,9% | 0,3% |  | 1,3% |  |
| **Ni -1 & Fe +1** | 18,96 | 19,98 | 14,12 | 15,06 |  | 12,1% | 22,4% | 63,9% | 0,3% |  | 1,2% |  |
| **Ni -1 & Zn +1** | 17,96 | 19,98 | 15,12 | 15,06 |  | 1,6% | 23,7% | 70,5% | 2,4% |  | 1,6% |  |
|  |  |  |  |  |  |  |  |  |  |  |  |  |
| **Cu +2** | 17,96 | 21,98 | 14,12 | 16,06 |  | 0,1% | 31,3% | 68,3% | 0,0% |  | 0,1% |  |
| **Fe +2** | 19,96 | 19,98 | 14,12 | 16,06 |  | 16,7% | 11,8% | 71,2% | 0,0% |  | 0,2% |  |
| **Zn +2** | 17,96 | 19,98 | 16,12 | 16,06 |  | 0,3% | 14,6% | 81,3% | 3,4% |  | 0,3% |  |
| **Ni -2** | 17,96 | 19,98 | 14,12 | 14,06 |  | 7,8% | 29,6% | 52,9% | 1,4% |  | 7,9% |  |

Santomera

|  | **Log Kc** |  |  |  |  |  |  |  |  |  |  |  |
| --- | --- | --- | --- | --- | --- | --- | --- | --- | --- | --- | --- | --- |
|  | **FeOHDMA** | **CuDMA** | **ZnDMA** | **NiDMA** |  | **FeDMA** | **CuDMA** | **NiDMA** | **ZnDMA** | **CoDMA** | **MnDMA** | **Free DMA** |
| **Experimental  after 168 hours** |  |  |  |  |  | *2,2%* | *67,6%* | *10,6%* | *9,5%* | *9,9%* | *0,3%* | *0,0%* |
| **unchanged** | 17,96 | 19,98 | 14,12 | 16,06 |  | 0,3% | 48,9% | 44,5% | 5,9% |  | 0,2% |  |
|  |  |  |  |  |  |  |  |  |  |  |  |  |
| **Change in Log Kc** |  |  |  |  |  |  |  |  |  |  |  |  |
| **Cu +1** | 17,96 | 20,98 | 14,12 | 16,06 |  | 0,1% | 69,0% | 28,6% | 2,1% |  | 0,1% |  |
| **Fe +1** | 18,96 | 19,98 | 14,12 | 16,06 |  | 3,2% | 47,5% | 43,5% | 5,5% |  | 0,2% |  |
| **Zn +1** | 17,96 | 19,98 | 15,12 | 16,06 |  | 0,2% | 37,6% | 35,0% | 27,0% |  | 0,1% |  |
| **Ni -1** | 17,96 | 19,98 | 14,12 | 15,06 |  | 0,8% | 62,9% | 22,5% | 13,1% |  | 0,5% |  |
|  |  |  |  |  |  |  |  |  |  |  |  |  |
| **Ni -1 & Cu +1** | 17,96 | 20,98 | 14,12 | 15,06 |  | 0,3% | 82,0% | 11,7% | 5,6% |  | 0,2% |  |
| **Ni -1 & Fe +1** | 18,96 | 19,98 | 14,12 | 15,06 |  | 6,9% | 60,6% | 20,4% | 11,5% |  | 0,4% |  |
| **Ni -1 & Zn +1** | 17,96 | 19,98 | 15,12 | 15,06 |  | 0,3% | 46,9% | 11,1% | 41,4% |  | 0,2% |  |
|  |  |  |  |  |  |  |  |  |  |  |  |  |
| **Cu +2** | 17,96 | 21,98 | 14,12 | 16,06 |  | 0,0% | 84,8% | 14,3% | 0,7% |  | 0,0% |  |
| **Fe +2** | 19,96 | 19,98 | 14,12 | 16,06 |  | 20,1% | 39,5% | 36,7% | 3,5% |  | 0,1% |  |
| **Zn +2** | 17,96 | 19,98 | 16,12 | 16,06 |  | 0,1% | 19,3% | 17,6% | 62,9% |  | 0,0% |  |
| **Ni -2** | 17,96 | 19,98 | 14,12 | 14,06 |  | 1,4% | 70,8% | 5,5% | 21,3% |  | 0,8% |  |

Xeraco L

Xeraco T

|  | **Log Kc** |  |  |  |  |  |  |  |  |  |  |  |
| --- | --- | --- | --- | --- | --- | --- | --- | --- | --- | --- | --- | --- |
|  | **FeOHDMA** | **CuDMA** | **ZnDMA** | **NiDMA** |  | **FeDMA** | **CuDMA** | **NiDMA** | **ZnDMA** | **CoDMA** | **MnDMA** | **Free DMA** |
| **Experimental  after 168 hours** |  |  |  |  |  | *57,1%* | *17,0%* | *9,7%* | *13,9%* | *1,0%* | *0,0%* | *1,3%* |
| **unchanged** | 17,96 | 19,98 | 14,12 | 16,06 |  | 0,3% | 5,3% | 91,3% | 2,6% |  | 0,0% |  |
|  |  |  |  |  |  |  |  |  |  |  |  |  |
| **Change in Log Kc** |  |  |  |  |  |  |  |  |  |  |  |  |
| **Cu +1** | 17,96 | 20,98 | 14,12 | 16,06 |  | 0,2% | 17,7% | 80,1% | 1,6% |  | 0,0% |  |
| **Fe +1** | 18,96 | 19,98 | 14,12 | 16,06 |  | 3,1% | 4,9% | 89,2% | 2,4% |  | 0,0% |  |
| **Zn +1** | 17,96 | 19,98 | 15,12 | 16,06 |  | 0,2% | 3,6% | 80,8% | 15,0% |  | 0,0% |  |
| **Ni -1** | 17,96 | 19,98 | 14,12 | 15,06 |  | 1,5% | 14,9% | 72,1% | 10,9% |  | 0,2% |  |
|  |  |  |  |  |  |  |  |  |  |  |  |  |
| **Ni -1 & Cu +1** | 17,96 | 20,98 | 14,12 | 15,06 |  | 0,9% | 32,3% | 59,3% | 6,9% |  | 0,1% |  |
| **Ni -1 & Fe +1** | 18,96 | 19,98 | 14,12 | 15,06 |  | 11,9% | 12,9% | 65,8% | 8,7% |  | 0,2% |  |
| **Ni -1 & Zn +1** | 17,96 | 19,98 | 15,12 | 15,06 |  | 0,7% | 8,9% | 50,5% | 39,4% |  | 0,1% |  |
|  |  |  |  |  |  |  |  |  |  |  |  |  |
| **Cu +2** | 17,96 | 21,98 | 14,12 | 16,06 |  | 0,1% | 34,1% | 64,5% | 0,9% |  | 0,0% |  |
| **Fe +2** | 19,96 | 19,98 | 14,12 | 16,06 |  | 18,3% | 3,1% | 76,9% | 1,4% |  | 0,0% |  |
| **Zn +2** | 17,96 | 19,98 | 16,12 | 16,06 |  | 0,1% | 1,4% | 54,3% | 43,8% |  | 0,0% |  |
| **Ni -2** | 17,96 | 19,98 | 14,12 | 14,06 |  | 4,6% | 25,2% | 40,3% | 28,8% |  | 0,6% |  |

SI-Figure 1: Comparison of the metal activities predicted with the multi surface approach and the CaCl_2_ extract method.
